# The experiences of parents raising children with developmental disabilities in Ethiopia

**DOI:** 10.1177/13623613221105085

**Published:** 2022-06-25

**Authors:** Bethlehem Tekola, Mersha Kinfe, Fikirte Girma Bayouh, Charlotte Hanlon, Rosa A Hoekstra

**Affiliations:** 1 King’s College London, UK; 2 Addis Ababa University, Ethiopia

**Keywords:** children, developmental disabilities, Ethiopia, parents, qualitative

## Abstract

The experiences of parents raising children with developmental disabilities have been widely researched, although most of this research comes from Western, high-income countries. In comparison, little is known about the lived experiences of parents of children with developmental disabilities in low- and middle-income countries and in Africa in particular. Parents’ lived experiences can provide insights into local conceptualizations of developmental disabilities, challenges children and their families face and their needs. This study explores the experiences of parents (14 mothers and 4 fathers) raising children with developmental disabilities in urban and rural Ethiopia using in-depth interviews. Cultural and religious beliefs played a role in the types of delays or differences in their child’s development that parents noticed early and the kinds of support they sought. Parents experienced stigma and lack of understanding from others. Their experiences regarding some of the challenges they faced such as lack of appropriate services varied based on where they lived (urban or rural). Single mothers especially were faced with multiple and intersecting struggles including poverty, stigma, and lack of social support. Implications for future research and interventions that aim to increase knowledge about developmental disabilities, tackle stigma and improve the lives of children and their families are discussed.

**Lay abstract:**

The experiences of parents raising children with developmental disabilities have been widely researched, although most of this research comes from Western, high-income countries. In comparison, little is known about the lived experiences of parents of children with developmental disabilities in low- and middle-income countries and in Africa in particular. We interviewed 14 mothers and 4 fathers in Addis Ababa and the rural town of Butajira to explore what life is like for parents caring for children with developmental disabilities in Ethiopia. Cultural and religious beliefs played a role in the types of delays or differences in their child’s development that parents noticed early and the kinds of support they sought. Parents experienced stigma and lack of understanding from others. Their experiences regarding some of the challenges they faced such as lack of appropriate services varied based on where they lived (urban or rural). Single mothers especially were faced with multiple struggles including poverty, stigma, and lack of social support. Implications for future research and interventions that aim to increase knowledge about developmental disabilities, tackle stigma and improve the lives of children and their families are discussed.

The experiences of parents raising children with developmental disabilities (DDs) including autistic children and children with intellectual disability (ID) have been widely researched (for a comprehensive review, see DePape & Lindsay, 2015 and Willingham-Storr, 2014). Most of this research comes from Western, high-income countries (e.g. Gray, 2003 in Australia; Woodgate et al., 2008 and Nicholas et al., 2016 in Canada; Downes et al., 2021 in France; Rafferty et al., 2020 in the United States, also well reflected in a recent review by Makino and colleagues, 2021). In comparison, there is a very limited number of published studies where investigators have examined the lived experiences of parents of children with DD in low- and middle-income countries (LMICs) in general, and in Africa in particular.

From LMIC, India has the largest literature on parents’ experiences of caring for their children with DD (e.g. Daley, 2004; Desai et al., 2012; Divan et al., 2012; Edwardraj et al., 2010; John et al., 2017; Minhas et al., 2015). In the majority of these studies, the influence of cultural beliefs and social norms on parents’ experiences was the main focus. In Africa, a small but gradually increasing number of qualitative studies have examined the experiences of parents raising children with DD (e.g. Aldersey, 2012; Cloete & Obaigwa, 2019; Gona et al., 2016; Masulani-Mwale et al., 2016; McKenzie & McConkey, 2016; Oti-Boadi, 2017). Most published studies on the experiences of caregivers raising autistic children and generally on autism in sub-Saharan Africa (SSA) comes from South Africa (Abubakar et al., 2016). Franz and colleagues (2017) reviewed six qualitative studies incorporating the perspectives of parents about autism from SSA (five from South Africa and one from Kenya). These studies covered topics such as parents’ perceptions on causes and treatment of autism (Gona et al., 2015), the challenges faced by parents (Olivier & Ah Hing, 2009), their coping strategies in the face of challenges (Alli et al., 2015) and the importance of professional support for parents (Fewster, 2015). The challenges faced by parents highlighted in these studies included lack of knowledge among professionals (Mitchell & Holdt, 2014), delays in getting diagnosis, and lack of social support and services (Mitchell & Holdt, 2014; Olivier & Ah Hing, 2009). More recent studies from SSA on parents’ experiences also discussed similar challenges including protracted diagnostic process (Reddy et al., 2019 in South Africa), financial and relationship problems (Cloete & Obaigwa, 2019 in Kenya), social isolation and stigma (Cloete & Obaigwa, 2019; Gona et al., 2016 in Kenya) and emphasized the need for increased resources and support for families. In relation to ID, a recent review of qualitative studies on caregivers’ experiences of raising a child with ID in Africa (Mkabile et al., 2021) suggests that similar issues (such as parents’ worries about the future, lack of services and stigma) are relevant also to families raising a child with ID.

In Ethiopia, there is a paucity of published literature on DD. The few published studies on DD conducted in Ethiopia highlighted lack of health and educational services for children with DD (Tekola et al., 2016; Zeleke et al., 2018), lack of awareness about DD (Tekola et al., 2016; Tilahun et al., 2016), unmet needs of families (Aldersey et al., 2020) and high level of stigma (Tekola et al., 2020a; Tilahun et al., 2016).

Building on these studies, this study aimed to explore the experiences of parents raising children with DD in urban and rural Ethiopia. Parents’ lived experiences can provide insights into local conceptualizations of DD (e.g. Desai et al., 2012; Edwardraj et al., 2010 in India), challenges children with DD and their parents face (e.g. Reddy et al., 2019; Woodgate et al., 2008) and their needs (e.g. Aldersey et al., 2020). However, as anthropological studies (e.g. LeVine, 1980) have shown parenting experiences can vary across cultures and settings. Thus, there is a need to explore parents’ lived experiences in a socio-cultural context.

Our focus on DD as a group is justified by the fact that in Ethiopia autism only comes to clinical attention when there is also substantial developmental delay, so all autistic children in this study either had a co-morbid formal diagnosis of ID or significant delay in one or more areas of development (language, motor or cognitive) in addition to challenges in social communication and social interaction. Our review of the LMIC literature also suggests that parents caring for an autistic child and/or a child with ID, to a large extent, shared similar experiences (e.g. stigma and lack of services). We have explored in detail the stigma experiences of the parents in another study (Tekola et al., 2020a). In this study, we explored the other aspects of the parents’ experiences including how stigma interacted with poverty, single parenthood, and DD in shaping some of the parents’ and their children’s experiences. Although the experiences of mothers raising children with DD have been extensively reported in the literature (e.g. Nicholas et al., 2016; Oti-Boadi, 2017), how multiple struggles reinforce one another in shaping the experiences of poor single mothers raising children with DD have not been sufficiently explored (Singh & Bunyak, 2019). There has also been inadequate response to support such women and their children. By incorporating both mothers and fathers living in urban and rural areas, which is not the case for previous studies from Africa which exclusively focused on the experiences of mothers living in urban areas (for exception, see Gona et al., 2016), this study will also provide a more comprehensive analysis of parents’ experiences.

## Methods

### Setting and context

The study was conducted in Addis Ababa (Ethiopia’s capital city) and the rural town of Butajira. In Addis Ababa, which has a population of over 5 million people, there are two child mental health clinics where DD can be formally diagnosed, and three centres for children with DD (Tekola et al., 2016). Some mainstream schools in Addis Ababa provide inclusive education programmes for children with DD, but provision is limited. In Butajira, which has a population of 260,000 people, there are no mental health services specific to children and no centres for children with DD. Throughout Ethiopia, the majority of children with DD remain without any formal diagnosis due to factors such as lack of knowledge and awareness, inadequate mental healthcare facilities and a severe shortage of trained personnel (Tekola et al., 2016; Zeleke et al., 2018).

### Participants and recruitment

Participants in Addis Ababa were identified via a child mental health clinic or schools for children with DD; participants in Butajira were identified by nurse-level health workers. Eighteen parents (nine from Addis Ababa and Butajira each) participated in the study. Fourteen of the eighteen participants were mothers, and four were fathers. In each site, we interviewed equal number of mothers (seven from Addis Ababa and Butajira each) and fathers (two from Addis Ababa and Butajira each). Four of the fourteen mothers were single mothers whose husband had left them after the birth of their child with DD. Except for four parents in Addis Ababa, all parents had at least one other child in addition to their child with DD. Ten parents were Orthodox Christians; seven were Muslims and one parent was protestant.

Most of the children (*n* = 14) had a formal clinical diagnosis of ID. Six children had autism as primary diagnosis; two of these children had a co-morbid formal diagnosis of ID, but the other four autistic children also had some degree of delay in one or more areas of development (language, motor or cognitive) in addition to challenges in social communication and social interaction. The children (13 boys; 5 girls) ranged in age from 4 to 9 years. Further participant details are presented in Table 1.

**Table 1.** Health and demographic information of parents and children who participated in this study.

Parents	Main diagnosis of child	Child’s age (years) and gender	Parent’s age (years) and parental role	Parent’s educational level
P1-U	Autism + ID	4; boy	41; father	12th grade^a^
P2-U	ID	9; boy	35; mother	5th grade
P3-U	ID	7; boy	37; mother	2nd grade
P4-U	Autism	7; boy	40; mother	No formal education
P5-U	Autism + ID	9; boy	43; mother	No formal education
P6-U	ID	8; girl	43; father	12th grade + 3 years further education
P7-U	ID	6; girl	39; mother	Basic literacy
P8-U	Autism	5; boy	30; mother	12th grade
P9-U	ID	7; girl	42; mother	11th grade
P1-R	ID and cerebral palsy	4; boy	29; mother	8th grade
P2-R	ID; ADHD	9; boy	25; mother	7th grade
P3-R	Autism	7; boy	30; father	6th grade
P4-R	ID	7; girl	35; mother	Degree
P5-R	ID	8; girl	27; mother	No formal education
P6-R	ID and epilepsy	8; boy	32; mother	6th grade
P7-R	Autism	9; boy	35; mother	8th grade
P8-R	ID	8; boy	30; mother	Basic literacy
P9-R	ID	9; boy	50; father	8th grade

ID: intellectual disability; ADHD: attention deficit hyperactivity disorder; U: urban (Addis Ababa); R: rural (Butajira).

a Completion of 12th grade is equivalent to completion of high school.

### Data collection

We interviewed all parents after or during their participation in a pre-test of the Ethiopian adaptation of the World Health Organization’s Caregiver Skills Training (CST) programme (Tekola et al., 2020b).

When we conducted interviews in Addis Ababa, our aim was to learn about parents’ experiences of taking part in the CST, and this is reflected in the questions included in the interview guide (see online Supplemental Appendix A-1). However, the in-depth nature of our interviews means that, in addition to their experiences regarding the CST, parents also talked about their experiences of raising children with DD. That is, we had given participants the opportunity to talk about issues that were important to them, not only those directly related to the questions in our interview guide (Braun & Clarke, 2013, p. 78). We also asked participants additional follow-up questions based on the issues which they shared with us, even when these were not directly related to the topics or questions listed in our interview guides. So, partly the actual questions we asked each of the participants depended on their responses.

After we coded the data from Addis Ababa, we observed that we had extensive information about parents’ experiences of raising a child with DD. All parents interviewed talked about their experiences of raising children with DD. Therefore, when we conducted the interviews in Butajira, in addition to their experiences of taking part in the CST, our aim was to learn more about the experiences of parents concerning raising children with DD. In our interview guide, we included questions relating to when and how they recognized delays or differences in their child’s development, the challenges they faced in relation to raising children with DD and generally about their family and social life (see online Supplemental Appendix A-2). Interviews were conducted in the local language of Amharic. The first author, who is bilingual in Amharic and English, interviewed almost all parents (17 parents). The remaining parent was interviewed by the second author. Prior to the in-depth interviews, demographic information was collected from all participants.

### Data analysis

All interviews were transcribed verbatim in Amharic and translated into English by researchers from Addis Ababa University who are bilingual in Amharic and English. All coding was done in NVivo 11 (QSR International Pty Ltd., 2015) on the English translations of the transcripts. We conducted thematic analysis guided by Braun and Clarke’s (2006, 2013, 2021, 2022) approach. We analysed the data in three steps since we collected the data from Addis Ababa and Butajira at different times (i.e. 7 months apart). In the first step, we analysed the data we collected from parents in Addis Ababa. This process started with the first author who conducted the interviews further familiarizing herself with the data. Then, she produced initial codes across all the data items. These initial codes covered parents’ CST experiences as well as their experiences in relation to raising children with DD. The first author led the analysis process and received input on the initial codes from the last author. In the second step, we analysed the data we collected from parents in Butajira. We followed similar steps as above. The first author generated initial codes across all collected data from Butajira. The first author then shared the initial codes and associated data extracts with the last and second authors to gain their input. In the third step, the first author combined initial codes from Addis Ababa and Butajira and sorted them into initial themes. These themes were developed after prolonged engagement with the coded data and data set.

We decided to combine the codes from the two study sites (rather than focus our analysis only on one of the sites) to gain a more rich and diverse accounts of the parents’ experiences as we believed where the parents lived (urban or rural) can influence their experiences. This was particularly important as most studies on parents’ lived experiences in Africa involved only parents who lived in urban areas as we explained in the introduction section. However, the small number of participants included in both this study sites (nine parents per site) as well as the flexible approach we used to collect data from the parents in the two sites (i.e. parents were not asked similar questions) means that we did not attempt to compare the views and experiences of the parents in the two sites. To do a meaningful such comparison, the participants in each site need to be representative of the larger population and the parents in each site should be asked similar questions (Lewis & Nicholls, 2013).

The initial themes we developed using codes from the two sites were revised and refined several times by the first author partly based on suggestions received from other authors before the final four themes and sub-themes were produced. Our analysis of the data from Addis Ababa was conducted inductively in the sense that when we coded the data from Addis Ababa, we were not engaged with the parents’ lived experience literature on DD. Thus, the codes we identified were strongly linked to the data (Braun & Clarke, 2006). However, before and after we collected the Butajira data, we engaged with this literature and other relevant works that may have influenced our analysis and interpretation of the data. So, in this sense, the analysis we conducted later was deductive (Braun & Clarke, 2006, 2021).

### Community involvement

The CST project which this study is part of benefitted from community stakeholders’ (including parents of children with DD) input throughout the project. However, children with DD and their parents did not participate in the analysis and interpretations of findings of this study.

## Results

From the parents’ accounts, we identified four main themes in relation to parents’ experiences of raising children with DD in Ethiopia. These are as follows:

1. Socio-cultural beliefs influenced recognition of and responses to delays/differences.
2. Nuanced and diverse family relationships and social life.
3. Multiple and intersecting struggles.
4. ‘My child is my jewel’: parents’ faith, positive outlook, and hope.

In the following, we present each of these themes with selected data extracts that vividly capture what the themes are about, the points we are demonstrating (Braun & Clarke, 2006) and the views of different parents (e.g. mothers, fathers, parents from urban and rural areas). The remainder of the data extracts that are related to each of these themes and sub-themes can be found in the Supplementary Materials (online Appendix B). Quotes from parents from Addis Ababa are denoted with ‘U’ to mean urban; quotes from parents from Butajira are presented with ‘R’ to mean rural.

### Socio-cultural beliefs influenced recognition of and responses to delays/differences

This theme is about how socio-cultural beliefs such as perceptions of disability and spirituality played a role in parents’ recognition of and responses to delays or differences in their child’s development. It contained two sub-themes: ‘noticing delays or differences’ and ‘parents’ responses’.

### Noticing delays or differences

Parents’ experiences varied regarding noticing delays or recognizing that anything was different in their child’s development. But, among the parents included in this study, there seemed to be no differences between parents in Butajira and Addis Ababa in terms of noticing delays or differences in their child’s development. Some parents in both sites reported that they had concerns about their child’s development at a very early age. Others reported that they were not aware of delays or anything different in their child’s development before a family or other people alerted them. A mother explained,

At first, I did not have any clue. It was around when she was a year and two months old when I took her to visit my family [that I came to know]. It was my mother, even earlier than this my mother and other family members had concerns. They used to say take her to the health centre. But I did not have any suspicion. [One day] When I took her to visit them, my mother took her to a private clinic in X town. I did not go with her; my mother took her with my sister. The doctor [at the private clinic] said to my mother that her suspicion was right, and my mother came back home and told me about it . . . (P4-R)

Some parents said they came to know about delays or differences in their child’s development from teachers after they put their child in school or pre-school. A mother explained,

I came to know about her condition after I put her in a kindergarten when she was four years old. I took her to her kindergarten, and I went to work. They called me and said, ‘take your child’. (P9-U)

It seems that parents noticed delays or differences in their child’s development early if their child showed motor developmental delays such as inability to walk and sit without support. As a father noted,

I noticed that my child had a developmental problem soon after he was born. He was very difficult to manage; he could not walk. I used a stick to help him walk. I realized that he had a developmental problem when it took him a long time to start walking. He started walking after he was three years old . . . (P9-R)

Some parental perspectives suggest that not speaking early was not considered atypical by the parents. In talking about the reaction of people regarding her autistic son’s delay in speaking, a mother in Addis Ababa, for instance, commented: ‘[people say] he would start talking. They would fill you with hope’. (P8-U)

The accounts of some parents of autistic children suggest that physical features of delayed development were given greater attention. Children who had atypical physical features were identified early and parents struggled to notice anything was different in their child’s development if their child had typical outward features. As two fathers commented,

. . . my child is very handsome . . . he was born in an Arab country, and he looks like his mother. Everybody likes him. His character is very good, and he is very friendly . . . my child can hear very well, and he can see properly; there is no problem other than his inability to speak . . . (P3-R)

The thing is, of course, my child has follow-up as they [health professionals] said he shows symptoms. If you see his face, my child is very handsome. He is very handsome. I am not exaggerating but he is handsome, has beautiful eyes and teeth and he also has a good physical structure. But he cannot speak . . . (P1-U)

The emphasis on physical features may also partly explains the lack of understanding about autism among the community as one of the parents in this study commented: ‘if it is not something like a [physical] disability such as hearing problem or not being able to walk, if it is autism, it is not known among people’ (P8-U).

### Parental response upon noticing delays or differences

Parents in both study sites explained that after they noticed delays or differences in their child’s development, they sought help from medical as well as traditional-religious sources. A father of a boy with ID and epilepsy who took his child to the hospital when he first noticed his epilepsy also said:

I went to a holy water place near X Hospital. We have stayed there for about two weeks with his mother. He has shown some improvement in terms of speaking. This is according to our belief. But the mental problem did not show any improvement. I have also taken him to a traditional healing place. I took him particularly for the epilepsy. But it did not help him much . . . (P9-R)

Similarly, one of the mothers in Butajira explained that her daughter with ID ‘started to walk eventually’ after she took her to a traditional healer. Parents’ accounts also indicated that their family and the community around them often advised them to take their child to traditional-religious places. A mother of an autistic boy explained:

Lots of people don’t openly tell you [about what they think regarding the child’s disability], but some people tell me to take him to the holy water place and the Muslims tell me to take him to the house of Quran. Be that as it may, in line with my religion I take him because anyone, even people who are healthy, need it. So, I take him. But they tell you that he will be healed that way. (P8-U)

### Nuanced and diverse family relationships and social life

This theme captures the nuanced and diverse nature of the parents’ family relationships and social life. Parents’ accounts suggest that, despite stigma and lack of understanding from others impacting on social relationships, not all parents were withdrawn from family relationships and social life completely and permanently. Depending on their current circumstances, the parents were either isolating themselves and their child from social life or they were putting their relationships and social obligations on hold (with the hope of resuming them later when their situation allows), or they were sustaining their family relationships and social life despite difficult circumstances.

Parents’ accounts suggest that they were often forced to distance their child and themselves from members of their family and generally from social life for lack of understanding from others. Parents said because people would not understand their child’s behaviour, they avoided taking their child to social places. A father explained,

I cannot take him to [the houses of] relatives and neighbours. If you ask me why? It’s because he can’t speak and when he tries to speak, he screams and he says like, ehh . . . ehh and the neighbours are not aware of such type of behaviour, they don’t have the understanding . . . (P1-U)

In addition to a lack of understanding about their child’s behaviour from others, in social places, parents also worried about their child’s safety which discouraged them from taking their child to social places. As a mother commented,

Nobody would tolerate his behaviour even for a day, nobody understands him . . . Even when I went to visit my family . . . I sat down and chatted, but my mind was absent, thinking that he might hurt himself. He was restless . . . (P3-U)

Faced with these challenges, mothers, who were often the primary care takers of their children with DD, were forced to withdraw from social life and stay at home alone with their child. In talking about the exclusion that she faced from her family members who saw her daughter’s disability as a curse and were embarrassed to be associated with her, a divorced single mother noted that she is prepared to give up her relationship with her family to be there for her child:

. . . [if] the worst comes I will be separated from my family. But I will not leave her [my daughter]. She is my child. She is my gift. That is because God will ask me. I am responsible for her . . . (P9-U)

The mother’s remark ‘she is my gift. That is because God will ask me’ suggest that she saw her responsibility towards her child as God given and as having repercussions if not fulfilled appropriately.

Parental perspectives also suggest that some have put relationships and social obligations on hold with the hope that when their situation improves, they would resume social life. A mother explained how her current situation prevented her from offering her condolence to her bereaved family members which is an important part of the expectations and obligations of social life in Ethiopia:

Now, I do not go to my family [to offer my condolences] when someone [in the family] dies. As a matter of fact, I do not go because there is no one who could look after my child [in my absence]. If you carry him and take him along with you, they [family members] will look at him as if he is another creation. So, if people hold grudges against me for not going to their houses [to offer my condolences] let that be. I prioritise my child. As long as I live, I prioritise my child. If things improve, I might have a social life just like any other people. (P2-U)

Another mother discussed how she recently resumed social life after she has put her relationships and social obligations (i.e. visiting those who gave birth and those who bereaved) on hold for some time:

. . . I only started to mix with people recently when she became older. I had completely stopped social life. Honestly, I had stopped going to visit those who gave birth and those who are bereaving . . . I had stopped social life to the extent that other people were starting to wonder what has happened to me. I would say ‘let them say whatever they want to say there is nothing more important than my child . . . I just stopped social life. But it was hurting me very much inside. I did not even go to the church . . . When I tried to manage her all people’s eyes were on me . . . I was isolated from social life. Now, when she became a bit older and when I tell her to stop, she is listening, and so now I go to [the church] . . . (P4-R)

The struggle parents were experiencing to maintain family relationships and to have social life in the face of misconceptions about their child’s disability from others was also featured strongly in the parents’ accounts. For some parents, this meant concealing their child’s behaviour and disability from others to continue interacting with other people in social spaces. A father explained,

So, to be honest, what I am doing is, I will take him [my child] to any place and when he tries to speak, I try to distract him by saying let us do this and that. That is because the more he speaks, the things that are speech for him may be odd for other people. His words are not common, so I will not tell that to neighbours. But I have not stopped him from mixing up with other people or play with them. (P1-U)

For others it means trying to live with their family in harmony despite misconceptions and lack of understanding about their child’s disability from their family members. A mother who started to live with her mother-in-law after periods of isolation explained:

P: My family could not understand that [my child’s disability] . . . I have quarrelled with my family lots of times. I even left their house and started to live by myself.

I: Are you referring to your family or your husband’s family?

P: Both my family and his family . . . I had left my mother as well as my mother in law’s house to live by myself saying that I do not want to live with both. But it is difficult to raise children without a family. (P1-R)

### Multiple and intersecting struggles

This theme illustrates how single mothers faced multiple struggles (such as poverty, stigma, and lack of social support) and how these struggles often intersected and reinforced one another:

I cannot explain it, there is a lot of burden. Even if we [mothers] are in pain, we tolerate it, there are lots of things. (P2-U)

The above quote from a single mother in Addis Ababa whose husband left her after the birth of their child with DD refers to the many struggles that some parents in this study experienced. Their struggle emanated from factors such as living in poverty, being a single parent, lack of understanding from others, experiencing isolation and stigma. In talking about the isolation and stigma that she faced from her family and the community around her the mother commented:

You will never be equal, whatever the case you will never be equal in your social life and family. I say this because I have witnessed many things. Even in your family a normal child and a child with disability will not be considered in the same way. That is because they do not have the understanding. They see it as an illness, they see it as something you brought because of your sin. There is pressure from family, neighbours and when you go on the street. (P2-U)

In her accounts, the intersectional experience of being poor, being a single mother, stigma and having a child with DD is visible. For her, stigma, and social isolation, which were experienced by many of the parents in this study, was not only about having a changed or strained relationship with family members and the community around her, but also a matter of survival. As she explained below,

My child and I are on our own; we do not have any other family. It is just Christ that we have. One day I was sick in connection with my heart problem. My child went on without eating food other than just bread for two days. I was only able to go to the shop and buy the bread; I could not prepare any other food. This was one of the times I was very sad and cried. (P2-U)

Another single mother in Addis Ababa who was struggling to bring income for her family of two described a similar view:

. . . Life is very difficult in this kind of situation [when you are excluded from your family and the community] with such kind of child . . . (P9-U)

The single mothers in this study were also compelled to bring income for their family at all costs even if this means compromising their children’s safety, and thus facing a moral dilemma. A single mother in Butajira explained that she had no choice but to lock her 8-year-old daughter with ID at home with her 10-year-old brother to go to the market where she was engaged in petty trading to feed her family of four and cover her house rent after her husband left her and their three children:

I leave the house in the morning [to go to the market] and return back home late. My first child, my son, looks after her [child with ID]. I used to lock the house on them and go to the market until her brother was old enough. She used to get out of the house and get lost. She used to wander around not giving much attention to whether a car is coming or not. For that reason, I was worried that I might lose her for a car accident so when I go to the market, I used to lock the door on her and her older brother. You know if I don’t go to the market, I can’t cover their expenses such as their bread . . . (P5-R)

Even for those single mothers whose child was old enough to go to school, bringing income for the family by working outside home was not easy. Lack of understanding and stigma means that single mothers were forced not only to isolate themselves and their children with DD from social life but also to abandon their means of livelihood. In talking about how she felt when a mainstream school refused to accommodate her daughter with ID while she was trying to work as a daily labourer a single mother in Addis Ababa recalled:

They [the teachers] did not even tell me to come and take my child calmly. You would not expect such kind of treatment from educated people. When I come hurriedly to the school, guess what she [the teacher] told me. She said the government will penalise us for keeping this kind of children at this school and she told me to take my child home. I still regret that I did not sue this teacher. That day my life became dark . . . do you understand if an educated person said this to you, there is no surprise that illiterate people stigmatise you. Then I take her [my child] home and for a year I did not take her out of the house . . . After a year when I took her out of the house, she used to get scared when she saw sunlight . . . (P9-U)

The mother’s account suggests that she was affected not only by the lack of appropriate support and service for her child with ID but also by the lack of awareness and the presence of stigmatizing attitudes among school staff. The mother was affected as much by the way the message was communicated as the message itself.

The accounts of parents suggest that generally there is a lack of understanding about DD among their family members, the general public and in professional and administrative services which often resulted in the parents and their child not getting the much needed practical and emotional support from others and institutions. A mother explained this lack of awareness:

. . . When you talk about autism, people would say ‘what is autism?’ For instance, the other day I went to our Kebele [smallest administrative unit] in connection with something and I asked the official at the Kebele to write me a letter stating that my child has autism. He asked me what autism is. I explained to him again and again [what autism is], but he could not even write it on a paper . . . (P8-U)

In addition to lack of understanding about their child’s disability and stigma, all the parents in this study were raising their children with DD in the context of inadequate or non-existent health and educational services. For the parents who lived in Butajira, getting appropriate support for their child was particularly challenging. Parents in Butajira explained that they are forced to travel to Addis Ababa or nearby town to get medical support for their children with DD. This puts financial pressure on these parents as they needed to pay for transport as well as services. In Butajira, where support for children with DD and their families is non-existent, schools were also missed not only as a source of educational support for children but also as a means of respite for parents. As a mother explained,

I would have been very happy if he was able to go to school, if he was able to learn like other children, learn how to hold his bag and lunch as this will have a big effect on him. It will reduce my responsibilities. There is nothing I want more than this. There is nothing that makes us [his father and I] happy than seeing him going to school and there is nothing we want more than this. (P2-R)

In response to our question about whether there is anything they would like to share or add after the interview, almost all parents emphasized the need to have schools or centres for their children with DD.

### ‘My child is my jewel’: parents’ faith, positive outlook, and hope

This theme highlights the hope and positive outlook expressed by parents which was often influenced by their faith.

Despite the multiple challenges that parents discussed in relation to lack of services, support, isolation and stigma, there were also parents in both study sites who interpreted their experiences in a positive light saying that their experiences made them stronger and more tolerant. Two mothers explained,

I think it helped me to be stronger. If other challenges come, I think I can handle them. The things I have experienced in relation to her [my daughter with ID] are difficult, they are making me a stronger mother . . . I am becoming stronger together with determination. She has made me stronger. (P4-R)

God has made us [parents of children with DD] more tolerant . . . Patience is not a simple thing. (P2-U)

Some parents also talked about how their children with DD positively changed their family relationships and their relationship with other people. A mother noted,

To be honest lots of people know me. They respect me. I have lots of respect because of my child. My child is my jewel. He is not something I am ashamed of . . . you have respect from lots of people, although those who do not have the understanding say bad things. People have different attitudes . . . (P2-U)

Many of them also remained hopeful. Spirituality and religion were important components of the hope expressed by parents:

A Christian is living with hope. I will never be hopeless. That is because I know what her situation was yesterday . . . Only God knows what will happen tomorrow. So, I am hopeful. (P9-U)

The hope expressed by parents also incorporated the need to accept their child’s disability as well as their belief about the power of God. As a mother explained,

I must accept my child’s condition. I cannot sell her. She is not cattle. I cannot exchange her. She is not a commodity. I just live with her praying to God. God has everything. (P9-U)

Parents’, especially mothers’, accounts of sacrifice and their determination to do anything for their child also have an element of hope for the improvement or cure of their child’s disability. A mother said,

I have sacrificed a lot for my daughter. Be it in terms of [changing] my religion. There is nothing that I have not done for her. (P4-R)

Another mother noted,

Everything is with my child. If there is anything which benefits my child, I will abandon everything else. (P8-U)

Similarly, in the quote below, a mother highlighted the power of God to change everything as well as her effort in bringing about improvement in her circumstances:

There is God and it will get better . . . We [parents of children with DD] are not going to stay like this. Our children may change with our effort. If a person puts an effort that person will achieve what they want. That is what we hope for . . .Wherever I go, I try to do things so he [my son’s condition] could improve. I do not hesitate [to do whatever necessary]. (P2-U)

Focusing on what the children were capable of doing rather than what they were not able to do also provided some parents positive outlook. As a mother expressed,

These children [children with DD] are wise. They are very wise. They know a lot. We do not need to see the fact that they cannot speak. They know a lot . . . What my son does is if I get sad, feel lonely . . . he says ema [my mom], hugs me and if I cry, he wipes my tears, consoles me, he knows . . . (P2-U)

## Discussion

The experiences of parents in this study varied regarding noticing delays or recognizing anything was different in their child’s development. Some parents in both study sites said they had concerns about their child’s development at a very early age. Others said they did not notice any delays or anything different in their child’s development before a family or teacher alerted them. This is consistent with previous studies on autism from high-income countries (see DePape & Lindsay, 2015) as well as LMIC (e.g. Daley, 2004) which highlighted variation in symptoms recognition and interpretation among parents.

In addition to parents’ lack of knowledge about DDs, cultural and religious beliefs seemed to play a role in the types of delays or differences in their child’s development that parents who had concerns in this study noticed early (see also Daley, 2004; de Leeuw et al., 2020). The parents noticed delays or differences in their child’s development early (before the age of 3 years) if their child showed motor developmental delays, for example, inability to walk on their own or sit independently. Some parents’ accounts suggest that not speaking early was not considered atypical. This observation is also reflected in an ethnographic study (Abdulwasie, 2007) which examined local expectations in relation to typical development of a child’s mental and physical behaviour in two neighbourhoods in Addis Ababa. The study found that not talking early (before age 5 years) is not necessarily considered as atypical. One of the adult informants in this study commented: ‘some children start to talk early, whereas others start late. Children who do not start talking do not necessarily have mental problems. If they are given time, they might start talking . . .’ (p. 42). The author reported that children are only considered to have a DD if at age 5 years they are not able ‘to demonstrate slightly their ability to communicate’ (p. 42). So, inability to speak seems to become a concern for parents only around school age. Abdulwasie (2007) further noted that girls were considered to talk, crawl and walk earlier than boys, a pattern also previously reported in India (Daley, 2004; Desai et al., 2012). Physical features of delayed development were given greater attention by the parents in this study. Children who had atypical physical features were identified early. However, parents struggled to notice anything was different in their child’s development if their child had typical outward features.

Although not directly addressed by the parents, their accounts suggest that the kinds of support they sought depended on their cultural and religious beliefs about the causes of delays or differences in their child’s development (de Leeuw et al., 2020; Kleinman, 1980). After they noticed delays or differences in their child’s development, parents tried different traditional-religious treatments such as holy water, praying and traditional healer (e.g. herbal cures) as well as medical help with the hope of getting an improvement or cure for their children’s DD. The parents’ accounts suggest that cultural and spiritual explanations such as seeing their children’s DD as a curse were common among their families, the parents themselves and the community at large (this is also discussed in Tilahun et al., 2016 and Tekola et al., 2020a in Ethiopia and Oti-Boadi, 2017 in Ghana).

The lack of awareness and understanding about DD strongly featured in the parents’ accounts in this study. Parents in both study sites spoke about lack of understanding about DD among their family members, the general public and the government/professionals. This was particularly discussed in relation to the parents’ stigma experiences. For instance, although in some instances school staff helped in recognition of their children’s DD, parents discussed about the stigma they experienced at schools. Parents’ accounts of their responses after they came to know about delays or differences in their child’s development suggest that a lack of understanding about DD extended to the parents themselves. These echo previous findings in Africa (e.g. Reddy et al., 2019) and elsewhere (e.g. Minhas et al., 2015 in South Asia), but the lack of awareness among administrative and government officials raised by the parents in this study has not been addressed by previous studies.

Several qualitative studies noted that, due to lack of understanding from others, parents raising children with DD may be isolated from social life (e.g. Oti-Boadi, 2017; Woodgate et al., 2008) and have strained family relationships (e.g. Fairthorne et al., 2014). The accounts of parents in this study suggest that the parents’ family relationships and social life were nuanced and diverse. That is, not all parents were withdrawn from family relationships and social life completely and permanently. Some parents have put some relationships and social obligations on hold with the hope that when their situation improves, they would resume their social life. Some parents indicated that they were forced to distance their child and themselves from members of their family and generally from social life because people would not understand their children’s DD. Others maintained some family relationships and social life despite lack of understanding and misconceptions about their child’s disability from others. In the absence of social welfare common in Western countries, like many societies in Africa, in Ethiopia, extended family members, neighbours and friends provide social, financial, and moral assistance to families at times of crisis such as financial hardships, sickness or death (Poluha, 2004). Reciprocity also shapes family and community relationships in Ethiopia. People are expected to invest in and nurture their relationships in order to receive or seek material and social support from others (Abebe, 2013). One way of doing this is fulfilling social expectations and obligations such as visiting those who gave birth and comforting those who have been bereaved, as mentioned by the parents in this study.

The parents’ accounts suggest that most of them were facing multiple struggles such as lack of appropriate services for their children with DD and financial difficulties, but often their experiences varied based on, among other things, where they lived (urban or rural). Although challenges related to education and health services were discussed by parents in both study sites, parents who lived in Butajira, where there is no centre or school for children with DD and limited health services, were affected the most. In many parents’ lived experience studies from Western, high-income countries, ‘navigating the system’ (e.g. DePape & Lindsay, 2015), is central to parents’ experiences of raising children with DD. Parents fight to get appropriate services for their children with DD. But like many countries in Africa, in Ethiopia services for children with DD are almost non-existent. In other words, there is no system to navigate.

Most of the parents in this study were struggling financially, but single mothers were affected the most. For single mothers, challenges such as stigma and lack of services were experienced on top of the acute financial difficulties they were facing. Most of the time they worried about how to survive: how to feed their family, fulfil basic needs and pay house rent. The intersection of single parenthood, poverty, stigma and DD also left single mothers and their children in this study without a means of livelihood. A divorced single mother of a daughter with ID in Addis Ababa described how because of stigma a school refused to accommodate her daughter resulting in her and her daughter staying at home without work and school, respectively. She could not get support from her family because she was isolated from them as they saw her daughter’s disability as a curse and were embarrassed to be associated with a child with ID.

Despite the multiple struggles in relation to lack of services, support, isolation and stigma, some parents in this study maintained positive outlook and hope. Similar to other studies from Africa (Gona et al., 2016) and elsewhere, particularly studies of African American parents of children with DD (e.g. Burkett et al., 2017), spirituality and religion were an important aspect of the hope expressed by the parents in this study. The parents’ commitments to care for their child (which was often seen as something presented from God, expressed in comments such as ‘she is my gift from God’) and their faith maintained their hope and meaning in life. Religion plays a central role in everyday life in Ethiopia, so explanations and expressions to life meaning are commonly associated with references to God.

## Implications for future research and practice

More ethnographic research is needed to have more nuanced understandings of what constitutes atypical delays or differences in this socio-cultural context. Specifically, (1) whether and why certain types of delays or differences are considered atypical than others by the parents, their family and the community around them; (2) whether factors such as gender of the child, socio-economic status of the parents’ and stigma influence such considerations; (3) the dynamic nature of socio-cultural beliefs means that longitudinal research is needed. Studies that focus on the perspectives and experiences of children with DD and that explore in more detail the experiences of fathers raising children with DD are also needed. Interventions that aim to increase knowledge about DD among parents and the community in general or tackle stigma should take into account these nuanced understandings.

Despite stigma impacting on social relationships, family relationships and social life were very central to the parents’ and their children’s lives’. In the absence of services, parents also relied heavily on their families for support. Increasing the knowledge of their families and the community around them about DD to decrease misconceptions and tackle stigma is crucial. In these efforts families can play a key role, for example, by being the main advocates for change (McConkey et al., 2016). Faith and religion also played an important role in the parents’ lives. Religious leaders can, therefore, be instrumental in tackling stigma by addressing misconceptions such as seeing DD as punishments for parents’ wrongdoing or as a curse from God.

This study also demonstrated that single mothers and their children were affected by multiple and often intersecting struggles such as poverty, stigma and lack of services. Interventions aimed at improving their quality of life, therefore, need to take into account these wider factors which may need community and structural level changes (Werner & Scior, 2016).

## Strengths and limitations

A strength of this study is that it incorporated participants living in a rural town, most of whom had a child who was only recently diagnosed with DD as part of the CST study. As such these families are similar to the majority of families with children with DD in SSA, most of whom remain without a formal diagnosis. Their accounts are likely to be more reflective of most African experiences than accounts from parents who have known their children’s DD diagnosis for a long time. A further strength of this article is that it highlighted not just distinct challenges faced by parents but also how many of these challenges are intertwined.

This study also has some limitations. When we conducted interviews in Addis Ababa, our aim was to learn about parents’ experiences of taking part in the CST, although we had given parents opportunity to talk about issues important to them beyond the CST training. A study that exclusively focused on their experiences of raising children with DD may have produced more rich and detailed accounts. What is more, the flexible approach we used to collect data from the parents in the two study sites (i.e. parents were not asked similar questions) means that we were not able to make meaningful comparisons between the views and perspectives of parents in the two sites. The fact that the parents were interviewed as part of the CST project, that is, all parents were enrolled in training, something unavailable to most families in Africa, could also be considered a limitation of this study as participation in the CST may have shaped parents’ perspectives. For example, the training may have influenced some parents outlook and hope about the future.

## Conclusion

This study highlights the varied experiences of parents raising children with DD in Ethiopia. Our findings suggest that poverty, single parenthood, stigma, and DD interacted with one another to shape the experiences of some parents raising children with DD in urban and rural Ethiopia. Despite diversity, parents raising children with DD in urban and rural Ethiopia also shared experiences in terms of ongoing struggle in relation to living in poverty, lack of appropriate support and services, stigma, and lack of understanding about DD from others. Parents, especially mothers who were primarily responsible for the daily care of their children with DD, prioritized the needs of their child by foregoing family relationships, social life, their work and their religion. Their faith and their commitments to care for their child helped parents to have a positive outlook and make sense of their lives.

## Supplemental Material

sj-docx-1-aut-10.1177_13623613221105085 – Supplemental material for The experiences of parents raising children with developmental disabilities in Ethiopia Supplemental material, sj-docx-1-aut-10.1177_13623613221105085 for The experiences of parents raising children with developmental disabilities in Ethiopia by Bethlehem Tekola, Mersha Kinfe, Fikirte Girma Bayouh, Charlotte Hanlon and Rosa A Hoekstra in Autism

sj-docx-2-aut-10.1177_13623613221105085 – Supplemental material for The experiences of parents raising children with developmental disabilities in Ethiopia Supplemental material, sj-docx-2-aut-10.1177_13623613221105085 for The experiences of parents raising children with developmental disabilities in Ethiopia by Bethlehem Tekola, Mersha Kinfe, Fikirte Girma Bayouh, Charlotte Hanlon and Rosa A Hoekstra in Autism
