# Supplementary material for: The experiences of parents raising children with developmental disabilities in Ethiopia
Source: Autism. 2022 Jun 25;27(2):539–51. doi: 10.1177/13623613221105085 (PMC13021001; doi:10.1177/13623613221105085)
Supplement: sj-docx-1-aut-10.1177_13623613221105085 – Supplemental material for The experiences of parents raising children with developmental disabilities in Ethiopia [file sj-docx-1-aut-10.1177_13623613221105085.docx]

**Appendix A: Topic guides**

1. **Topic guide for interview with caregivers in Addis Ababa post CST pre-pilot testing**

Family ID_______________ Participant ID: ____________________

Family Member:

 mother  father

 grandmother  grandfather

 sister/stepsister  brother/stepbrother

 other (specify) __________________________

Date: ___________________

Location: ________________

A. **Introduction**

Interviewer to say his/her name and where they are from.

We are here to learn more about your experiences taking part in skills training for caregivers of a child who develops slowly. The information you give us will help us to further adapt the training as necessary, so that it meets the needs of families in Ethiopia. While we are talking, we are recording what is said with this tape recorder so that we can remember all of the information. Everything we learn from people will be put together so that we have an idea of the views and experiences of many different families. There are no right or wrong answers.

B. **Interview guide**

[Start tape recording from here, start tape recording with saying the family ID number, but not the name of the caregiver]

- Ask about experiences with programme, both positive and negative.

Please tell me about your experience of the programme

What did you like about the programme?

What didn’t you like about the programme?

- Explore the usefulness of the programme to the caregivers.

Tell me about how relevant the programme was to your circumstances.

How useful was the programme? In what ways was it useful?

Did the programme help you to feel more confident? In what ways?

Did the programme help you to feel less stressed? In what ways?

Did the programme help you to improve your child’s skills? In what ways?

Was the programme useful in ways that you did not foresee or expect? Please tell me in what ways?

- Explore usefulness of CST components and strategies.

What part of the programme was most useful to you? Why?

Tell me about your experience of the home visits? What were the positive and the negative aspects?

How did you find the videoing?

Tell me about your experience of group sessions? What were the positive and the negative aspects?

Which strategy that you learned was the most useful? Why?

- Ask for suggestions to improve relevance and usefulness of CST content.

What else would you have liked the CST programme to address?

What other important messages do you think need to be conveyed to caregivers of children with developmental delays or developmental disorders?

- Explore acceptability of programme content.

How did you find the messages and stories that were used in the CST sessions? How much did those messages and stories fit with your situation?

- Ask about experience and difficulties with implementing the strategies at home and suggestions to make homework more feasible.

What made it hard to implement the intervention with your family? What helped to make it easier?

- Explore barriers with attending the programme and suggestions to improve attendance and

reduce drop out.

What made it easy/hard for you to attend the training sessions? Any suggestion to make it easier to attend?

- Explore programme impact on families.

What kind of impact has the training had on you? How about on your child? How about other

members of your family? How about on relationship among family members

- Explore if the programme implementation has triggered any change in the community.

How do you find getting out and about in your community? Has the programme affected that in any way? Please tell me about that. [Probe about awareness, accessing support, stigma]

- Explore adverse effect on caregivers, families and communities.

Did you or your family experience any difficulty or challenge or discomfort in relation to attending the CST programme?

Have you experienced any negativity from your community since the start of the CST programme implementation? [If so, ask for suggestions to avoid this in the future.]

- Ask for any additional suggestions and thank participants.

Is there anything else you would change about the programme?

Are there any other thoughts or comments you would like to share with us?

**ID code □□□□□**

**2. Topic guide for interview with caregivers of a child with a developmental disorder in Butajira**

**A. Introduction**

Interviewer to say his/her name and where they are from.

We are here to learn about your thoughts and experiences regarding raising a child with a developmental disorder (a child who develops slowly). While we are talking, we are recording what is said with this tape recorder so that we can remember all the information. Everything we learn from people will be put together so that we have an idea of the views and experiences of many different families. There are no right or wrong answers.

**B. Background**

| S. N | First of all, we just need some background information. | | | |
| --- | --- | --- | --- | --- |
| 1 | What is your gender? | Male | 1 | GEND |
|  |  | Female | 2 |  |
| 2 | What age are you now? | _________ years | | AGE |
|  | Date of Birth (if known) | [ ] | |  |
|  | Today’s date | [ ] | |  |
| 3 | How much formal education have you received? | No formal education [ ] | | EDU |
|  |  | Completed grade [ ] [ ] | |  |
| 4 | What is your marital status? | Married | 1 | MARSTAT |
|  |  | Single | 2 |  |
|  |  | Divorced | 3 |  |
|  |  | Widowed | 4 |  |
| 5 | Do you practise a religion? |  |  | RELIG |
|  |  | Orthodox Christian | 1 |  |
|  |  | Protestant | 2 |  |
|  |  | Catholic | 3 |  |
|  |  | Muslim | 4 |  |
|  |  | Other (please specify): __________ | 66 |  |
| 6 | What is your occupation? | Housewife | 1 | OCCU |
|  |  | Student | 2 |  |
|  |  | Unemployed | 3 |  |
|  |  | Farmer | 4 |  |
|  |  | Government employee | 5 |  |
|  |  | Private employee | 6 |  |
|  |  | Trader | 7 |  |
|  |  | Other (please specify):______________ | 66 |  |
| 7 | What is your relationship to the child who has problems with development we will be talking about today? | Mother | 1 | RSWCDD |
|  |  | Father | 2 |  |
|  |  | Brother | 3 |  |
|  |  | Sister | 4 |  |
|  |  | Grandmother | 5 |  |
|  |  | Grandfather | 6 |  |
|  |  | Aunt | 7 |  |
|  |  | Uncle | 8 |  |
|  |  | Other (please specify): ____________________________ | 66 |  |
| 8 | Do you live with the child? | Yes | 1 |  |
|  |  | NO | 2 |  |
| 9 | How old is the child with problems with development? | ____________________________ |  |  |
| 10 | Is this child a boy or a girl? | Boy | 1 | GEND |
|  |  | Girl | 2 |  |
| 11 | How many children do you have? |  |  | NOC |
| 12 | Do any of your other children have developmental problems? | Yes | 1 | DP |
|  |  | NO | 2 |  |

If yes, please give details about their problems:

**C. Interview guide**

**[Start tape recording from here, start tape recording with saying the participant ID number, but not the name of the caregiver]**

**[Use Prompts only when a respondent does not readily answer questions]**

- When did you recognize that your child has a developmental disorder? (When did you recognize that your child is developing slowly?)
- What did you do when you came to know that your child is developing slowly?

Prompts:

- Did you seek medical help?
- Did you get traditional treatment (e.g. going to holy water place)?
- How did your family respond to learning that your child has a developmental disorder?
- What are the challenges of raising a child with developmental disorder?

Prompts:

- Managing child’s difficult behaviour
- Lack of understanding from others (e.g. when out in public places)
- Social isolation
- Financial burden
- Difficulty getting appropriate health service
- Difficulty getting appropriate education
- How do you deal with these challenges?
- What is helping you to deal with these challenges?

Prompts:

- Having a supportive/understanding family, relatives, friends and neighbours?
- Religion?
- Has having a child with DD changed your family’s life? If so, how?

Prompts:

Social:

- In terms of relationship among family members (with husband/wife, with siblings, among siblings)?
- In terms of relationship with neighbours?
- In terms of relationship with relatives?
- In terms of relationship with friends?

Psychological:

- Increased stress?
- Do you feel like your relationship with other people has changed because of having a child with DD? If so, how?
- Do you include your child with DD in family events and gatherings? If so, how? How do other family members react? If no, why?
- Do you feel that people treat you or your family differently because of your child's developmental disorder?

If yes, can you please elaborate on the nature of other people’s reactions?
