# Supplementary material for: The experiences of parents raising children with developmental disabilities in Ethiopia
Source: Autism. 2022 Jun 25;27(2):539–51. doi: 10.1177/13623613221105085 (PMC13021001; doi:10.1177/13623613221105085)
Supplement: sj-docx-2-aut-10.1177_13623613221105085 – Supplemental material for The experiences of parents raising children with developmental disabilities in Ethiopia [file sj-docx-2-aut-10.1177_13623613221105085.docx]

**Quotes from participants relating to the four themes**

| Participants | **Socio-cultural beliefs** **influenced recognition of and responses to delays or differences** | **Nuanced and diverse family relationships and social life** | **Multiple and intersecting struggles** | **“My child is my jewel”: parents’ faith, positive outlook, and hope.** |
| --- | --- | --- | --- | --- |
| Parent 1-U | The thing is, of course, my child has follow-up as they [health professionals] said he shows symptoms. If you see his face, my child is very handsome. He is very handsome. I am not exaggerating but he is handsome, has beautiful eyes and teeth and he also has a good physical structure. But he cannot speak…  …my child’s behavior and that of other children’s is definitely different. I mean, it is not that I know about this thing more than you, but each child’s behavior is different. Some children might be easy to manage, and other children might be difficult to manage. Those one or two cases that I saw here are different from my child. My child can get close to you if you treat him with love, but other children may not calm down even if you tried to calm them down… | I cannot take him to [the houses of] relatives and neighbours. If you ask me why? It’s because he can’t speak and when he tries to speak, he screams and he says like, ehh...ehh and the neighbours are not aware of such type of behaviour, they don’t have the understanding…  So, to be honest, what I am doing is, I will take him [my child] to any place and when he tries to speak, I try to distract him by saying let us do this and that. That is because the more he speaks, the things that are speech for him may be odd for other people. His words are not common, so I will not tell that to neighbours. But I have not stopped him from mixing up with other people or play with them.  …I don’t care about what people say or don’t say. But, regarding my child, I need him to grow up freely. I don’t hide him from people just because people might say something about him, I try to make him to mix up with people. Of course, my wife doesn’t want him to get close to people, I don’t want to deny that. That is because she is afraid that people might say something about him and exclude him. But I always want him to be free. Sometimes, we may argue because she doesn’t want him to get out of the house, but I want him to get out of the house because he should experience everything, the sun, the cold weather so that it won’t be a new thing for him. But people still have a problem. | …It would have been nice if something can be done for our children. For example, it might be a school or a place for them to stay and to develop their mind…it might be some kind of school…I went to two or three places, but they said, “we will call you when we have free space” … But what if there is no space? Am I supposed to keep my child at home?  …the burden of life. For example, people need to work hard just to get something to eat…they work hard to carry on their life…when such kind of things come together, you may not focus on your child…  ...peoples understanding is very low… they have lack of awareness...  I am not talking with neighbours or other people that my child has this type of case, I don’t want to let them to blame me. When they ask me why my child is not talking, I tell them that it takes time to get an improvement. I tell them that, it’s not possible to have a radical change. That’s how I try to clarify things for them but it’s still challenging… | …A child can reach a better place if the parents take care of him properly. If the parents understand their child’s need and support him/her properly, the child will be very successful …I can assure you this. Because we might face different burden at our home and if we are able to pass that and effectively do what we can, we can really be successful… |
| Parent 2-U | …my son couldn't walk until he was seven. It happened slowly, I used to carry him on my back, hold his hand to walk together, he started walking slowly. I used to carry him on my back, he couldn't walk until he was seven. He used to walk a little and then fall… since his bones were weak, it is after a long time and with patience that he was able to walk. | Now, I do not go to my family [to offer my condolences] when someone [in the family] dies. As a matter of fact, I do not go because there is no one who could look after my child [in my absence]. If you carry him and take him along with you, they [family members] will look at him as if he is another creation. So, if people hold grudges against me for not going to their houses [to offer my condolences] let that be. I prioritise my child. As long as I live, I prioritise my child. If things improve, I might have a social life just like any other people.  On the road, on transport people don't usually understand. There is also a big problem in social life… We got into a bus once and he [my child] snatched a hat from an older person, this person did not know about my son's condition. When my son took his hat, that person took the hat back and hit him. Another person, I think he knows about such children, asked that older person if it was appropriate to do that [hit the child] and they got into an altercation…. And all these has a big influence on social life… | I cannot explain it, there is a lot of burden. Even if we [mothers] are in pain, we tolerate it, there are lots of things… Eh... I am raising my son without a father ... Eh... I have a heart condition but now thanks to God it is getting better. I am getting better ... Eh... When I get stressed, it relapses but now I am getting a little better. I am taking the medication… Still now I have a lot of burden. X has seen my house, it's very small. We can't move like we want to and do things, also it is only me and my son, there is no one else. My son's relationship is only with me…  Today we are here [for our children] but what about tomorrow? [At some point] everyone passes away. Let alone [passing away] leaving such kind of children, normal children who lose their mother will not have a good life…I pray to God to take him before me. I always beg God day and night. I do not want my son to beg for help [after I have passed away].  My child and I are on our own; we do not have any other family. It is just Christ that we have. One day I was sick in connection with my heart problem. My child went on without eating food other than just bread for two days. I was only able to go to the shop and buy the bread; I could not prepare any other food. This was one of the times I was very sad and cried.  You will never be equal, whatever the case you will never be equal in your social life and family. I say this because I have witnessed many things. Even in your family a normal child and a child with disability will not be considered in the same way. That is because they do not have the understanding. They see it as an illness, they see it as something you brought because of your sin. There is pressure from family, neighbours and when you go on the street. | … I was hopeless … eh I used to think that my child can’t mix with other people. Now my child has come out and I also came out, many people have seen us. We met with many parents. We shared with each other our pains and many other things and I believe we are going to bring about many changes …  There is God and it will get better ... We [parents of children with DD] are not going to stay like this. Our children may change with our effort. If a person puts an effort that person will achieve what they want. That is what we hope for ...Wherever I go, I try to do things so he [my son’s condition] could improve. I do not hesitate [to do whatever necessary].  God has made us [parents of children with DD] more tolerant… Patience is not a simple thing.  To be honest lots of people know me. They respect me. I have lots of respect because of my child. My child is my jewel. He is not something I am ashamed of…you have respect from lots of people, although those who do not have the understanding say bad things. People have different attitudes….  These children [children with DD] are wise. They are very wise. They know a lot. We do not need to see the fact that they cannot speak. They know a lot…What my son does is if I get sad, feel lonely…he says ema [my mom], hugs me and if I cry, he wipes my tears, consoles me, he knows…  ... everything happens with God's will… |
| Parent 3-U | …Before we knew about his condition there was no place that we did not take him. He was always sick and there were times that we did not have money for his treatment. If you add this on top of the money that we spend on his food, it was very difficult. So, I used to get upset. God knows….  I was affected a lot. I did not expect that he will be like this. God does according to his will. Who can interfere with what He does?  I faced many things before we know his case… I was following my child’s treatment at private health facilities but after my uncle heard about my son, he advised me to go to X [government run hospital] and he supported me in this regard. | Nobody would tolerate his behaviour even for a day, nobody understands him… Even when I went to visit my family… I sat down and chatted, but my mind was absent, thinking that he might hurt himself. He was restless…  I: How is the attitude of people in other places?  P. One day when we go to X Hospital when he [my child with DD] was wrestling, one person warned me not to go with my child on a taxi next time.  I. Who was that person?  P. He was the driver assistant and the person sitting next to me asked me [about my child] and I told him that he has a problem. Then, he was about to fight with the assistant saying that he has a problem, he has autism… Then, after this person got off from the taxi, the assistant was saying to me you were about to make me hit by that person. I tried to ignore him. I have faced such kind of problem. | I need to pay house rent. I struggle a lot financially, caring for him needs lots of money. That is because he gets sick often. There are times that I work for two days and then do not go to work. I need to take him to hospital and because of that it is very difficult. I do not work enough days. My husband is also a daily labourer.  When he [my son with ID] wrestles with me after I get back home tired from work, I used to say, ‘God, what have I done?’ There were times that I cried a lot…it is very challenging. | …I will praise the lord one day. One-day God will improve his behavior [referring to his restlessness] … |
| Parent 4-U | …my child couldn’t talk…he was restless. I have four children. He is the youngest… He couldn’t wear his cloth in the previous times and couldn’t say anything. | I don’t meet up with neighbours. I don’t mix up with anybody. I sit at home with the child. When I go with the child to places, he does not sit down. He disturbs me. I don’t mix up with people. I don’t meet my relatives anymore. I don’t mix up with anybody. I don’t mix up with neighbours. I don’t mix up with anybody. When my [other] children come [from school] I open the door for them and then close the door. | I used to work previously but after I gave birth to him, I sat at home leaving my work, I sat at home for the last seven years since I gave birth to him… he is my last child, and I am living in this way. My husband doesn’t get work most of the time but when he manages to work, he will go to his work early in the morning and return to house at night.  In pervious times my neighbours supported me and looked after my children when somebody died and I needed to go somewhere but after the birth of this child, as I told you, I have distanced myself from everybody. I don’t talk with anyone. After his condition, no one will talk with me and even they don’t greet me…They came and visited me in the previous times but now there is no one who will talk with me but am living thanks to Lord… My mother is living in a rural area. I have no father and I have no other relatives here. |  |
| Parent 5-U | My child has speaking problem. He is restless. He understands some orders. He was restless in the previous times and he used to run here and there but now he is showing changes. When you order him to do something, he is reluctant to do it…it is very difficult to explain his condition… He gets disturbed and becomes restless when there are many people. | My commitment is with my child and I don’t have any relationship with my neighbours except exchanging greetings. I don’t know anything about my neighbours, and no one discuss with me about my child’s condition. As you know the thing about our community is, they talk behind your back when you go out with the child or when the child does something. But that does not stop me. I don’t feel ashamed. I go with him to the shop. I go with him to the church. I take him everywhere I want. People can say different things, but I don’t give much attention to that. | …I left my work because of my son…  There is no one who would look after our child including our families due to their behavior. Handling such kinds of children is difficult…. let alone my neighbors my mother is not willing to care for such kinds of children. These children are difficult to handle due to their behavior and even our mother is not willing to care for them. My [other] children are looking after my child because they have the awareness, but they will not live with me forever. They will lead their life in the future.  There isn’t much awareness about this condition in our country. Now the community knows about HIV/AIDS, about those who are unable to see and hear…we live in Addis Ababa [the capital city] but these children don’t have their own centers.  We need now support with school because this problem is not only our child’s problem but there are many mothers who are crying because of this problem and I am not the only mother who have interest to send my child to school but there are many children who have this interest…we tried for two years still we couldn’t find a place for him. If there are better conditions, we will not face such problems… This will help both the parents and the children. The families will not worry, and the children will get freedom and their mind will be changed.  As I told you before, I don’t have any relationship with my neighbors except greetings. I don’t have deep relationship with them. Nobody knows about this child’s condition and even if they know, no one will tell you about it… In the previous times, this community excludes people with HIV/AIDS. Similar to that, the community doesn’t want these children [children with developmental disability] to touch and push their children. I don’t worry about this. He [my son] plays with his siblings at home. | …It is very difficult, but God knows about the future.  There are many parents who have such kind of children. If there are better things everybody will be willing to openly talk about them and search for solution. For example, HIV patients used to hide themselves in the previous times but now everybody wants to openly talk about it because many things have been done about HIV and there are good things now. If we do good work regarding this condition, I am sure our children will reach great places. I have great hope. This is what I want to say.  … I have hope that things [talking about stigma] may change in the future and better things will come. |
| Parent 6-U | I used to wonder how I would get through the future with her condition, for how long would I handle it as she gets older, I wouldn’t be able to handle it, what would I be able to do…  ...I was just frustrated and asked for how long this could go on, I asked God things like that I mean for how long she would continue to be like this, there are many things that are unpleasant. For example, when people say your daughter is like this and this, I used to feel sick inside… |  | They [the school] put her [child with ID] in different classes every day. If she goes to Grade 1 today, I will find her in grade 4 tomorrow. When this happens who takes the responsibility for the risk [that could happen] to my child? Most of the time she spent her time in staff room. May God forbid if something happens to her who takes the responsibility…? | I have sacrificed a lot for my daughter. Be it in terms of [changing] my religion. There is nothing that I have not done for her. |
| Parent 7-U |  | P. My neighbours may say many things, but I didn’t get upset and cry like the previous times because I took the training.  I. What did they say, for example?  P. as you know, people say many things. They consider it as a curse, and they say many other things and give many suggestions. Due to this, I cried many times before I took this training but now, I didn’t cry after I took this training. I didn’t say anything when they say different things and I prefer to be silent. This is the result of the training. | P: …we should train them [children with DD] how to protect themselves because they are growing.  I. When you say how to protect themselves what do you mean? Can you please explain that for me?  P. For example, my child does not fear strangers and follow and go with them even to far places and if they snatched her, she would not say anything, and she will not come back home. I wish that we [parents] get lessons in this area |  |
| Parent 8-U | …he [my son] can't express himself or speak. He used to push me and point to things. I would usually bring things for him…If he wants to eat, he will bring the food to me. He wouldn't communicate in any way. We just wouldn't communicate. He wouldn't talk to me….  When I came to know about my child’s condition, I have not heard about this kind of condition before, about autism. And I used to get scared a lot. I did not have a good feeling. I did not sleep. I did not have a good life. I did not know what I needed to do, and I was confused.  … because I didn’t understand my child’s behaviour, I used to think that he was spoilt, or I was a weak mom. When I get angry with his behaviour, I used to think that it is because I am a bad mom ….  There aren't that many people that openly tell you. You don't know. Lots of people don't openly tell you [about what they think regarding the child’s condition], but some people tell me to take him to the holy water place and the Muslims tell me to take him to the house of Quran. Be that as it may, in line with my religion I take him because anyone, even people who are healthy, need it. So, I take him. But they tell you that he will be healed that way.  … [people say] he would start talking. They would fill you with hope… |  | Most people do not know about autism…if it is not something like a [physical] disability such as hearing problem or not being able to walk, if it is autism it is not known among people. When you talk about autism, people would say ‘what is autism?’ For instance, the other day I went to our Kebele [smallest administrative unit] in connection with something and I asked the official at the Kebele to write me a letter stating that my child has autism. He asked me what autism is. I explained to him again and again [what autism is], but he could not even write it on a paper…  …my son doesn't go to school. I had enrolled him to a school, and I was happy. Because he was learning, and he was getting out of the house…But because they were unable to handle him, they told me to take him home. After he stopped school, I was very sad. I wish they [children with DD] have school of their own. If they could get out of the house and stuff. Seeing that would make me happy. I was very happy when he went to school. And he even got used to it. He will get up in the morning and pick up his bag and uniform. It took some effort to make him stop doing that… | Everything is with my child. If there is anything which benefits my child, I will abandon everything else. |
| Parent 9-U | I came to know about her condition after I put her in a kindergarten when she was four years old. I took her to her kindergarten, and I went to work. They called me and said, ‘take your child’.  She knows everything… her problem is speaking. She couldn’t speak up until she was four years old but after she was four years old, she started using some words like water. | P.…I am excluded by my family including my mother.  I. What did they say?  P. Their problem is embarrassment. They told me not to bring my child to their house during daytime [when people can see her] and they told me to bring her to their house during the night-time [so that nobody can see her]. But only Satan moves at dark. We, children of God, will move in the daytime. They are worried about their dignity.  You told me about your relationship with you family earlier. How is your relationship with your neighbours?  P. It is unthinkable.  I. Unthinkable?  P. Yes, it is unthinkable. You know why it is because I am living in a community which does not understand you. My daughter, for example, fears fight, she is calm and doesn’t touch anyone and she also doesn’t want to be touched by others, but the community sees her in another way. They think she will hit and push their children. There is exclusion. They don’t understand you. In this kind of situation, it is difficult for your mind.  …[if] the worst comes I will be separated from my family. But I will not leave her [my child with DD]. She is my child. She is my gift. That is because God will ask me. I am responsible for her… | There is a huge pressure. There is exclusion starting from my family and there are very difficult situations, but I don’t have a choice but to accept that. There is a problem when we go on the road and when people see us and even in our family. You will be excluded eh... especially from my family, nobody accepted me including my mother. Because of this, I am not living in the community. I am living only with my child…  The awareness level of the community is very low, and a lot needs to be done. They don’t share their thought with you. They talk about you behind your back and because of that, you will be forced to exclude yourself from them. That is because…we are not living with educated people. They believe in the curse and they give different explanations. Due to this and to protect your mind you will exclude yourself. It has a huge impact. It is very difficult.  She was four years old. I took her to a kindergarten, and I went to my work… They [the teachers] did not even tell me to come and take my child calmly. You would not expect such kind of treatment from educated people. When I come hurriedly to the school, guess what she [the teacher] told me. She said the government will penalise us for keeping this kind of children at this school and she told me to take my child home. I still regret that I did not sue this teacher. That day my life became dark...do you understand if an educated person said this to you, there is no surprise that illiterate people stigmatise you. Then I take her [my child] home and for a year I did not take her out of the house… After a year when I took her out of the house, she used to get scared when she saw sunlight… | A Christian is living with hope. I will never be hopeless. That is because I know what her condition was yesterday…Only God knows what will happen tomorrow. So, I am hopeful.  I must accept my child’s condition. I cannot sell her. She is not cattle. I cannot exchange her. She is not a commodity. I just live with her praying to God. God has everything. |
| Parent 1-R | Since birth you could tell from his physical features. On top of that when he starts to grow, the way he does things compared to other children. That means when other children do things that he cannot do. I came to know when he is slow at doing things compared to other children.  I went to X Hospital, I went to many places, I haven’t sat down… | P: My family could not understand that [my child’s condition] …I have quarrelled with my family lots of times. I even left their house and started to live by myself.  I: Are you referring to your family or your husband’s family?  P: Both my family and his family…I had left my mother as well as my mother in law’s house to live by myself saying that I do not want to live with both. But it is difficult to raise children without a family.  …my mother, I have told you, she used to say take him [my child] to the back of the house when someone comes to the house. And my biggest fight with my mother was: ‘why do you say that; it is not good’. They only think about the family [name]…. | …once in a taxi, I was holding him [my child] when I get into the taxi. It was an old lady, she looked shocked and said, ‘in the name of the father.’ I asked her what was wrong because I was shocked [by her reaction] too. She said that this thing [my child’s condition] was a curse and that I should ask my family. I cried a lot until I couldn’t talk …  …for instance, when I take out [name child], just, ask me who doesn’t turn around to look at him, everyone turns around to look at him, that is just it, I just suggest to them to watch where they are going so that they won’t fall down, that is what how I mean, there is no one who doesn’t turn to look at him  …the thing that I always ask myself is what is the effect of the community’s attitude on the child later. I swear to Allah I am very worried. I am worried that this may lead him to become mad…  …if we are three, me, my older one and my youngest, the older one gets uncomfortable because they [people] stare at his youngest brother, he asks me why they stare, and he gets upset. I tell him to ignore them…But I feel it inside me, I can imagine how he is feeling…  …truly, people are killing what is left of my energy. They are just making me be ashamed when I decide to take him [my child] out… I am starting to believe that in rural areas the mothers who hide their children behind closed doors are right. That is because this [mothers hiding their children] is a result of the public’s responses. Tomorrow, when I take my child out what could happen, will be worse…  …he needs to play but he is made not to play, he needs to jump, without getting any of childhood stuff, his time will pass locked up in a room. His story shouldn’t be this. His peers’ stories shouldn’t end this way, the government should, I don’t know, there needs to be a situation where other stakeholders get involved with our issues…there needs to be more done for our children. There needs to be a center for our kids. |  |
| Parent 2-R | I came to know [about his condition] when I tried to put him in school. They told me that he is beyond their capacity and that he has a condition. They said he needs a different school. When two to three schools said similar things, I was sad and returned home with my child. But I did not stay home silently, I started looking for solutions everywhere. I even went to Addis Ababa… | The attitude of other people, because x [my child] can listen but can’t talk, they say, why doesn’t your child talk. My neighbours used to say my child is Duda [tongue-tied]. They were saying, did you give birth to a Duda? They were teasing me. They were making fun of me saying her oldest child is like this. They were saying I must have done something [to cause the child’s condition]. They were saying all of this in my presence, but I used to pretend that I was not listening but cry when I got home…  My family sees it as a curse… this still hurts me… because he is my first child, I tried to ignore what people say. But I say to myself Allah gave me this child. May He be my vindicator and give me | We can’t afford to fulfil all his needs. We have other children and as you can see, we live like this. I have tried to contact the city administration but by the time I brought up the issue to them they said there is no one working on this case so they can’t help me. I also went to the Education Bureau and they said that my child has special needs so he needs a special needs teacher, but I couldn’t find any teacher. The one I find was a teacher for those unable to see and his name is X, he teaches in secondary school. He really supported me. He calmed me down and encouraged me saying this kind of children need patience and it is not a curse. My relatives used to think that it’s some kind of curse and I used to feel really bad about it.  I would have been very happy if he was able to go to school, if he was able to learn like other children, learn how to hold his bag and lunch as this will have a big effect on him. It will reduce my responsibilities. There is nothing I want more than this. There is nothing that makes us [his father and I] happier than seeing him going to school and there is nothing we want more than this.  …What I am worried a lot about is who is going to look after my child like I do if I die. Nobody will look after him. People are avoiding him even when I am here. If I die nobody will care about him… |  |
| Parent 3-R | …my child is very handsome… he was born in an Arab country and he looks like his mother. Everybody likes him. His character is very good, and he is very friendly…my child can hear very well, and he can see properly; there is no problem other than his inability to speak…  I have taken him both to X and Y hospital… I have hope that he will start to speak when Allah allows it. | I think the problem does affect my social life… I try to make him play with kids, but when I see that they talk and play together but he can’t do that, and I sometimes feel bad when they neglect him. When I feel that way, I take him out and try to have him play with leaves… | Last time, x [a protestant church] was registering children to give education. And I was told to bring him [my child] as well. I took him hoping that he will spend time with other children and be able to learn. Even though I am a Muslim I don’t have a problem with the religion… I only wanted my child to spend time with other kids and be able to learn. But once they realised that he can’t speak they refused to register him. I was willing to pay them as much as I can afford but they said no while all his friends got registered. I felt sad that day…  When his mother and I take him to his grandmother’s area when they [people] say ‘is he better now’. He has nothing wrong except that he does not speak…his mother and I understand him, what he wants. Other people may not understand him and think that he is ill. When they say ‘is he better now’ that makes me sad.  …sometimes while he is staying at his Grandmother’s house my nephew’s call him names because he can’t speak, and I don’t like that, so I get into a fight with them and ask them why they keep calling him names, and sometimes people like neighbors or brother in laws come and try to speak to him in a sign language and I hate that…  Working on increasing awareness on this area can help a lot because some people might think that it is because of our sin and that has a major impact on the child. I sometimes ask him what people were saying about him and he told me that they call him names and I felt really bad, when children are given such names it has its own effect. I raised my kid to be as free as possible and, but such things have impact. | I sometimes feel bad when I hear other children speak, but I know this is Allah’s will, not only the child but I also complain to Allah sometimes, but I will never give up on him, I still have hope that he will start speaking.  …He has no sin at all, he didn’t cause any problem or may be this is because we have sinned a lot that is why Allah did this to us, or maybe he might forgive him I will never give up I think all is because of Allah. |
| Parent 4-R | At first, I did not have any clue. It was around when she was a year and two months old when I took her to visit my family [that I came to know]. It was my mother, even earlier than this my mother and other family members had concerns. They used to say take her to the health centre. But I did not have any suspicion. [One day] When I took her to visit them, my mother took her to a private clinic in X town. I did not go with her; my mother took her with my sister. The doctor [at the private clinic] said to my mother that her suspicion was right, and my mother came back home and told me about it…  I did not know what it is. I have not seen it before. It was not in my family. And what can I tell you, that day [the day I have been told about my child’s ID] I said, ‘what have I done wrong?’ I said why? What have I done?’…  When I first knew about my child’s condition, I used to not talk about it. When neighbours asked me about my child, I used to say she has a heart problem and did not say she has a developmental problem. I considered it as something bad happened to me. I did not talk about it. It is only recently that I started to talk about it… after I became aware, I started to talk with confidence. | …I only started to mix with people recently when she became older. I had completely stopped social life. Honestly, I had stopped going to visit those who gave birth and those who are bereaving… I had stopped social life to the extent that other people were starting to wonder what has happened to me. I would say ‘let them say whatever they want to say there is nothing more important than my child’. I would not openly say to them that this is because I have such kind of child. I just stopped social life. But it was hurting me very much inside. I did not even go to the church because she was difficult to handle there. When I tried to manage her all people’s eyes were on me…I was isolated from social life. Now, when she became a bit older and when I tell her to stop, she is listening, and so now I go to [the church] … | There is a problem. I don’t like it when people pity her. I say to them: ‘what is wrong with her?’ and I even try to fight with them. On the taxi and on the street when people stare at her I say: ‘what are you looking at?’ May God give them understanding…  It is people, their attitude, which make you sick when you try to take your child out. This is maybe because of lack of awareness but their attitude is not good.  It is very difficult. When you take your child to the church you feel embarrassed. I used to think as if I am different. I used to say: ‘what have I done wrong’…  For the first three years [after giving birth] I abandoned my job for which I studied many years and stayed at home. People were saying ‘did you put your [education] documents under your pillow’. [These were] People who did not know about my situation. I have not shown them my daughter. People abandon their work and sit at home even after having a normal child, if you have a child like her think about how challenging it is. It is very difficult. | I think it helped me to be stronger. If other challenges come, I think I can handle them. The things I have experienced in relation to her [my daughter with ID] are difficult, they are making me a stronger mother…I am becoming stronger together with determination. She has made me stronger.  I have sacrificed a lot for my daughter. Be it in terms of [changing] my religion. There is nothing that I have not done for her.  Thank God, she [daughter with DD] has increased love among us [me and my husband]. What I see in some families is rift [between husband and wife]. But here the love she has for her father is special. He is also good. Thank God. God has not disappointed me in this regard. When he gives me a child like her, he gave me a good husband… my family supports me. My mother lives a bit far from here, but she supports me morally. When I take my child to her place, I feel uncomfortable thinking that people may see her. My mother and sisters are not like that. They take her out… |
| Parent 5-R | I: When did you notice delays in your child’s development?  P: I knew since birth. Her body was not strong. She just sat down; she did not try to walk. When I tried to train her to walk, she could not do that. Her legs and arms were weak like a leaf; she just sat down. She sat down [without walking] for almost 3 years… |  |  | Deep down in my heart I always say ‘how can I improve her [condition]?’ may Allah do that. I pray saying ‘you are the only one who have the ability I don't have the ability’… |
| Parent 6-R | P: I knew about it after two years  I: After he turned two years old?  P: Yes  I: How did you know?  P: After that he was not like others. He was late to roll around and walk. The others walk on their ninth month. But he was delayed. I became aware about his situation after that. Before that some people told me that “some children are like that. They have late development.” They said like that and I also thought like that. Then I took him to X hospital and started following up his case there. He has been following up there up to now. |  | It would have been great if there was a place that he [8 years old boy with ID] spends the day and come home later. You can consider me as a prisoner. I cannot work or carry out anything. |  |
| Parent 7-R | I have taken him to X [hospital] several times. They told me many times that he is anaemic. I have been giving him milk to drink. After that they said he needs surgery… They were saying he may or may not be cured. I got scared thinking that what if he died. Thus, I refused and took him back home. I left X [hospital] and started following up at Y [non-governmental organisation providing care for neurological problems], here in Butajira…But he didn’t show any improvement…Then, I took him to a private health facility located in Addis Ababa.…after that I got desperate. I got desperate and took him to a holy water... I was taking him there and I observed several changes in my child… | It is very difficult. May God help us. If you have such kind of child, you need to be very strong…it is only recently that I started to mix with people…. up until he is 6 years old, he pees and poops on himself. I did not mix with people thinking that they may be disgusted. I felt like all people walking on the street are only looking at me…. | …because my child is like this my husband was even asking me to leave. We were about to divorce. He was even saying: ‘I don’t want to see your eyes take your child and leave’. I was not telling this to my family. I was just crying… |  |
| Parent 8-R | One day I took him to the health centre for an illness. The lady at the health centre knew [that he has a developmental problem]. She said, ‘why are you not taking action, why don’t you take your child to the hospital?’ But I took him to the health centre for a different kind of illness. When she said that [to me] I did not have the financial capacity to take him to the hospital. So, for a while I did not take any action…. let alone a hospital in Addis Ababa I don’t have the financial capacity [to cover the expenses] here [in Butajira]. I took a while until I arranged that… |  | For him no to mix up with other children, there are some foolish people who think that his condition is contagious. We feel sorry inside. We would be happy if he could mix up and play with other children. He doesn’t hit people, even if they hit him, he doesn’t hit back, um, um, he doesn’t hit, in fact kids younger than him hit him…  Some people say that my child’s problem came because I have laughed [at other people] … it’s not that I have laughed, only Allah knows the reason why it came. |  |
| Parent 9-R | I noticed that my child had a developmental problem soon after he was born. He was very difficult to manage; he could not walk. I used a stick to help him walk. I realized that he had a developmental problem when it took him a long time to start walking. He started walking after he was three years old…  I went to a holy water place near X Hospital. We have stayed there for about two weeks with his mother. He has shown some improvement in terms of speaking. This is according to our belief. But the mental problem did not show any improvement. I have also taken him to a traditional healing place. I took him particularly for the epilepsy. But it did not help him much...  I: …how did the other family members respond when they came to know about your child’s disability?  P: They told us to come clean if we have any worship of other gods in the family. I have noticed that people’s awareness around this issue is very poor.  I: Are you referring to people living around here?  P: People living around here as well as our families. My mother-in-law told me to look for a worship of other gods in my family and she said that is why the child is not having any teeth or why he is not speaking. I told her that my family doesn’t have such things, we don’t have any of these things. I have other children and they are all fine except this child. So, I have nothing to look for in my family is what I told her. I hate my mother-in-law still now because she said those things... |  | Interviewer: Tell me about the challenges of raising a child with developmental disability?  Participant: oh, it is very difficult. It requires forbearance. If there are no other responsibilities it may be possible but if you have other responsibilities it is difficult to look after and raise this child. He fights with other children. He frequently fights and cries…he has difficult behaviour. He would not listen to anybody except doing what he wants. If you try to intervene, he will throw things and break things…he also frequently fights with other children and people would say ‘manage your mad child’…This kind of child would cause stress for the parent and mother. You would not discipline him because he has mental illness, and he does not know [what he is doing]. … So, it is difficult…it has challenges.  …both my wife and I needed to work to bring income for the family. But we worry until we come home thinking that he may follow strangers and disappear. There was child theft in our area recently and we were worrying a lot about his safety… |  |
